# Borax affects cellular viability by inducing ER stress in hepatocellular carcinoma cells by targeting SLC12A5

**DOI:** 10.1111/jcmm.18380

**Published:** 2024-05-23

**Authors:** Ceyhan Hacioglu, Didem Oral

**Affiliations:** ^1^ Faculty of Pharmacy, Department of Biochemistry Düzce University Düzce Turkey; ^2^ Faculty of Medicine, Department of Medical Biochemistry Düzce University Düzce Turkey; ^3^ Faculty of Pharmacy, Department of Pharmaceutical Toxicology Düzce University Düzce Turkey

**Keywords:** borax, endoplasmic reticulum stress, hepatocellular carcinoma, SLC12A5

## Abstract

Hepatocellular carcinoma (HCC) presents a persistent challenge to conventional therapeutic approaches. SLC12A5 is implicated in an oncogenic capacity and facilitates the progression of cancer. The objective of this investigation is to scrutinize the inhibitory effects of borax on endoplasmic reticulum (ER)‐stress and apoptosis mediated by SLC12A5 in HepG2 cells. Initially, we evaluated the cytotoxic impact of borax on both HL‐7702 and HepG2 cell lines. Subsequently, the effects of borax on cellular morphology and the cell cycle of these lines were examined. Following this, we explored the impact of borax treatment on the mRNA and protein expression levels of SLC12A5, C/EBP homologous protein (CHOP), glucose‐regulated protein‐78 (GRP78), activating transcription factor‐6 (ATF6), caspase‐3 (CASP3), and cytochrome c (CYC) in these cellular populations. The determined IC50 value of borax for HL‐7702 cells was 40.8 mM, whereas for HepG2 cells, this value was 22.6 mM. The concentrations of IC50 (22.6 mM) and IC75 (45.7 mM) of borax in HepG2 cells did not manifest morphological aberrations in HL‐7702 cells. Conversely, these concentrations in HepG2 cells induced observable morphological and nuclear abnormalities, resulting in cell cycle arrest in the G1/G0 phase. Additionally, the levels of SLC12A5, ATF6, CHOP, GRP78, CASP3, and CYC were elevated in HepG2 cells in comparison to HL‐7702 cells. Moreover, SLC12A5 levels decreased following borax treatment in HepG2 cells, whereas ATF6, CHOP, GRP78, CASP3, and CYC levels exhibited a significant increase. In conclusion, our data highlight the potential therapeutic effects of borax through the regulation of ER stress in HCC by targeting SLC12A5.

## 1 INTRODUCTION

Hepatocellular carcinoma (HCC) stands out as one of the most prevalent primary liver malignancies, with its incidence and mortality rates steadily escalating in recent years.^1^ This gastrointestinal cancer exhibits a high occurrence and is responsible for over 830,000 cancer‐related deaths annually.^2^ Consequently, there exists an urgent imperative to explore novel strategies and drug targets for the treatment of HCC. Cancer cells, including those implicated in HCC, undergo metabolic changes that impact processes such as cellular viability and metabolic homeostasis.^3^ These alterations frequently disrupt the redox balance in HCC cells, thereby influencing diverse signalling mechanisms such as endoplasmic reticulum (ER) stress and cell death pathways, ultimately culminating in carcinogenesis.^4^ Despite the reported promising treatment approaches, the overall prognosis and poor median survival for HCC patients have yet to witness significant improvement.^5^

ER is an organelle found in eukaryotic cells, comprising a network of interconnected membrane vesicles. It assumes a critical role in functions such as lipid synthesis and protein folding, albeit being susceptible to various toxic influences.^6^ Increasing research indicates that ER stress is intricately associated with the regulation of apoptosis.^7^ Glucose‐regulated protein‐78/binding immunoglobulin protein (Grp78/Bip), activating transcription factor 6 (ATF6), protein kinase R‐like ER kinase (PERK), and inositol‐requiring kinase‐I (IRE‐1) are proteins linked with the unfolded protein response (UPR) in cells.^8^ These proteins are pivotal components of ER stress and contribute to the UPR mechanism.^9^ When the ER fails to maintain homeostasis during the UPR, it triggers cell death through the induction of pro‐apoptotic program mechanisms within the cell.^10^ ATFs play a crucial role in modulating the expression levels of various proteins involved in cell death pathways, including the pro‐apoptotic protein C/EBP homologous protein (CHOP or DDIT3), which serves as a critical determinant of cell fate.^11^ This process involves the phosphorylation of Eukaryotic Initiation Factor 2 alpha (eIF2α) by the activated protein kinase RNA‐like endoplasmic reticulum kinase (PERK), resulting in a rapid reduction in overall protein synthesis and preferential translation of specific genes.^12^ These gene products may aid tumour cells in adapting to changing conditions. Prolonged activation of the UPR axis may paradoxically trigger apoptotic cell death. Multiple lines of evidence suggest the pivotal involvement of PERK/eIF2α/ATF4/CHOP in tumour progression.^10^ It has been reported that ER stress induced by various compounds contributes to apoptosis by increasing caspase‐3 activity and enhancing CHOP expression in various cancer cell types.^13^ ER stress induces HCC aggressiveness and drug resistance, as well as correlates with poor patient prognosis, and pharmacologically targeting ER stress mediators has yielded promising results.^14^ Therefore, the activation of ER stress in cancer cells may represent a potential therapeutic avenue in HCC treatment.

The solute carrier (SLC) family constitutes the largest group of transmembrane transport proteins.^15^ These proteins play a vital role in facilitating the transportation of various substances crucial for maintaining cellular viability across cell membranes. Additionally, they contribute significantly to the communication between intracellular and extracellular environments.^16^ Despite this, there has been limited reporting on the effects of SLC12A5 in human cancers. A previous study demonstrated that high overexpression of SLC12A5, along with increased expression of SRY‐box transcription factor 18 (SOX18), promoted the proliferation and metastasis of bladder urothelial carcinoma cells.^17^ Furthermore, elevated levels of SLC12A5 have been documented in ovarian carcinoma and colorectal cancer cells, where they induce carcinogenesis and correlate with diminished survival outcomes.^18^, ^19^ Additionally, elevated levels of SLC12A5 have been associated with a poor prognosis and the promotion of tumorigenesis in HCC.^20^ However, investigations into the effects of SLC12A5 on tumour viability are still in their nascent stages.

Boron, a trace element commonly distributed in soils, oceans and freshwater, primarily manifests in forms such as borates, boric acid, and borax.^21^ Due to its role in regulating redox balance and cellular metabolism, researchers have extensively explored the impact of boron on signalling pathways using in vitro and in vivo models.^22^ In our previous investigations, we discovered that various boron compounds exert influence on cell viability by modulating diverse signalling pathways in cancer cells.^23^, ^24^, ^25^ Kırlangıç et al. found that combined treatment of borax with 5‐Fluorouracil resulted in a significant increase in apoptotic cell death on human colorectal adenocarcinoma cells.^26^ Furthermore, an earlier study suggested that boric acid exhibits a regulatory effect on ER stress‐related pathways in prostate cancer cells.^27^ According to gene ontology analysis, borax was shown to inhibit cell growth in HepG2 cells by downregulating genes associated with stress response, apoptosis, inflammation, and proliferation.^28^ Similarly, another study found that various boron compounds exhibited anti‐cancer effects by inducing increased levels of lipid peroxidation alongside oxidative damage in HepG2 cells.^29^ Additionally, Hung and colleagues reported that boric acid could accelerate tumour cell death by selectively damaging tumour cells and hepatoma blood vessels at hepatoma sites in in vivo HCC models.^30^ Kahraman and Göker found that boric acid treatment inhibited AKT phosphorylation in HCC cells, suppressed colonization and migration, and induced various cell death pathways.^31^ Despite prior demonstrations of boron's regulatory effects on cancer cells across various metabolic pathways, its specific impact on ER stress in HCC cells remains elusive.

In this study, we investigated the effects of borax on SLC12A5 in HepG2 human HCC cells and HL‐7702 human normal liver cells. Furthermore, we examined whether borax treatment in HepG2 cells would inhibit cell proliferation by promoting ER stress through the regulation of SLC12A5 expression. The study entailed analysing the levels of biomarkers associated with ER stress and apoptosis, including ATF6, CHOP, GRP78, cleaved caspase 3 (CASP3), and cytochrome c (CYC), in HepG2 and HL‐7702 cells subsequent to borax treatment.

## 2 MATERIALS AND METHODS

### 2.1 Cell culture

In the experimental processes, HL7702 and HepG2 cell lines were obtained from the American Type Culture Collection. Cells were cultured in Dulbecco's modified Eagle's medium (DMEM; 51435C, Sigma‐Aldrich) containing 10% fetal bovine serum, 200 mM L‐glutamine, penicillin–streptomycin (100 U/mL–100 μg/mL; 15140122, GIBCO) in the culture medium. The cells were seeded onto 75 cm^2^ plates (Corning Life Sciences) and cultured at 37°C in a humidified chamber with 5% CO_2_ and 95% air until reaching approximately 80% confluency.

### 2.2 Cell viability assay

Borax (HT1002, Sigma‐Aldrich) was solubilized in either 0.1% dimethyl sulfoxide (DMSO; 20688, Thermo Scientific) or complete cell culture medium at 25°C. Subsequently, the stock solution (0.4 M) was further diluted to attain the necessary concentrations for various assays. On the day of the experiment, the stock solution was filtered and prepared fresh. Before treatment, HL7702 and HepG2 cells were seeded in 96‐well plates and incubated in culture medium for 24 h until approximately 80% confluency. Subsequently, the cells were treated with borax concentrations ranging from 0.5 to 64 mM for up to 72 h. Following, measurements were made at 570 nm using the cell viability analysis kit (TOX1‐1KT; Sigma‐Aldrich) based on 3‐(4,5‐dimethylthiazol‐2‐yl)‐2,5‐diphenyltetrazolium bromide (MTT) analysis, in accordance with the manufacturer's instructions. The untreated control group's cell viability was designated as 100%, and the viability of cells subjected to borax treatment was computed relative to this control group. The cell viability curve was constructed using the calculated percentage values of cell viability for each concentration of borax, and the concentrations corresponding to 25% (IC25) and 50% (IC50) inhibition were ascertained from this curve.

### 2.3 Cell proliferation assays

To assess the impact of borax on the proliferation of HepG2 and HL‐7702 cells (1 × 10^5^), seeded in 12‐well plates, we employed a commercially available 5‐bromo‐2′‐deoxyuridine (BrdU) kit (2750; Sigma‐Aldrich). HepG2 and HL‐7702 cells were treated with borax concentrations determined according to MTT results for 24 h. The proliferation rate of cells was then analysed colorimetrically based on BrdU incorporation assay, which detects DNA synthesis during cellular replication, following the manufacturer's instructions. Absorbance values were analysed at 450 nm using a microplate reader (BioTek).

### 2.4 Morphological and nuclear analyzes

To visualize changes in cellular structure, HepG2 and HL‐7702 cells were seeded at a density of 1 × 10^3^ cells per well and treated with borax for 24 h. After the treatment, the cells were gently washed with phosphate‐buffered saline to eliminate any remaining compounds. Subsequently, observation and analysis of alterations in cellular structure were conducted using an inverted microscope, specifically the Oxion Inverso equipped with a CMEX‐5 Pro camera.

Nuclear differences in borax‐treated HepG2 cells were performed by 4,6‐diamidino‐2‐phenylindole (DAPI; D8417, Sigma‐Aldrich) staining. The cell was incubated in DAPI solution for 30 min at 25°C in the dark, and then the dye was removed. Next, the nuclear structures in the cells were visualized by fluorescence microscopy with nuclear counterstaining.

### 2.5 Cell cycle analysis

HL7702 and HepG2 cells were seeded at a density of 2 × 10^5^ cells per well in 12‐well plates and treated with various concentrations of borax for 24 h. After the treatment period, the cells were harvested in the culture medium. To preserve cellular structures and prevent further changes, the cells were fixed with a 4% paraformaldehyde solution. To analyse the cell cycle distribution, the fixed cells were stained with propidium iodide (PI), which binds to DNA and allows visualization of the cell cycle stages. PI staining distinguishes cells in different phases of the cell cycle, including G1 (gap phase 1), S (DNA synthesis phase), G2 (gap phase 2) and M (mitosis phase). The intensity of fluorescence emitted by the stained cells was measured using the Muse® Cell Analyser.

### 2.6 Biochemical analysis

To determine the levels of SLC12A5, ATF6, CHOP, GRP78, CASP3 and CYC, ELISA assay kits (LS‐F65961, LS‐F36737, LS‐F8872, LS‐F6280, SEA626Hu and SEA594Hu, respectively) were utilized following the manufacturer's instructions for each kit. In the experimental protocol, HepG2 and HL‐7702 (at a density of 1 × 10^5^ cells) cells were placed in 96‐well plates and exposed to concentrations of borax for 24 h. Subsequent to the incubation period, the absorbance of each sample was measured using a microplate reader at the wavelength specified in accordance with the instructions provided in the ELISA assay kit.

### 2.7 Quantitative polymerase chain reaction (qPCR)

Total cellular RNA was extracted using TRIzol (Invitrogen, 12594025), and cDNA from total mRNA was synthesized using the SuperScript™ IV One‐Step kit (Invitrogen, 12594025). All samples were subjected to triplicate analysis using the Step One Plus™ Real‐Time PCR System (Applied Biosystems, Foster City, CA, USA). The qPCR was conducted under the following conditions: a preincubation step for 20 min at 60°C followed polymerase activation for 10 min at 95°C, an amplification process included an initial denaturation step at 95°C for 15 s followed by 40 cycles of annealing and extension at 60°C for 1 min. The primers used for the amplification reactions of SLC12A5, ATF6, CHOP, GRP78, CASP3 and β‐actin were as follows: SLC12A5 forward primer: 5′‐GCG GAA GCT AAC TAG ACC‐3′, reverse primer: 5′‐CGT ACC ATC TGC GTA CCG‐3′; ATF6 forward primer: 5′‐CAG GGT AGC ATC TGA TCA GAG‐3′, reverse primer: 5′‐GAC GCT CAA TTG AGG GTT AGC‐3′; CHOP forward primer: 5′‐AGC GAC TTC AGG GTC GTT GCG‐3′, reverse primer: 5′‐AGG CAT GGA GCT GCA ACG TCC‐3′; GRP78 forward primer: 5′‐GCT GAG GAA TCC TGC GTG AGC TGC‐3′, reverse primer: 5′‐CGG ACC TTG CGA GCT ATT GGA AGG‐3′; CASP3 forward primer: 5′‐GGC AGC CAG GGA GTC GAA GTC AGC‐3′, reverse primer: 5′‐GGT CGA ATG CCG ACG GAA CCA GTC‐3′. The determination of relative gene expression was carried out utilizing the 2^−ΔΔCT^ method, with normalization performed against the internal control β‐actin.

### 2.8 Western blot assay

The protein levels of SLC12A5, ATF6, CHOP, GRP78 and CASP3 in HepG2 and HL‐7702 cells were evaluated through western blot analysis. This method was carried out systematically, adhering to the procedural details outlined in our prior study.^23^ The antibodies utilized for analysis included SLC12A5 antibody (PA5‐78544, Invitrogen), ATF6 antibody (PA5‐20216, Invitrogen), CHOP antibody (PA5‐88116, Invitrogen), GRP78 antibody (PA1‐014A, Invitrogen), cleaved CASP3 antibody (PA5‐77887, Invitrogen), and β actin antibody (PA1‐183, Invitrogen). To visualize the protein bands, an enhanced chemiluminescence kit (34579, Thermo Scientific) was employed in accordance with the manufacturer's protocol. Western blot analyzes were quantified using ImageJ.

### 2.9 Statistical analysis

Statistical analyses were performed using GraphPad Prism 8 software. The data, collected from three independent experiment replications, are presented as mean ± standard deviation (SD). To compare the experimental groups, one‐way analysis of variance (ANOVA) and two‐way ANOVA were employed, with statistical significance determined at a *p* < 0.05.

## 3 RESULTS

### 3.1 Borax exerted regulatory effects on cell viability and proliferation in both HL‐7702 and HepG2 cells

**FIGURE 1:**
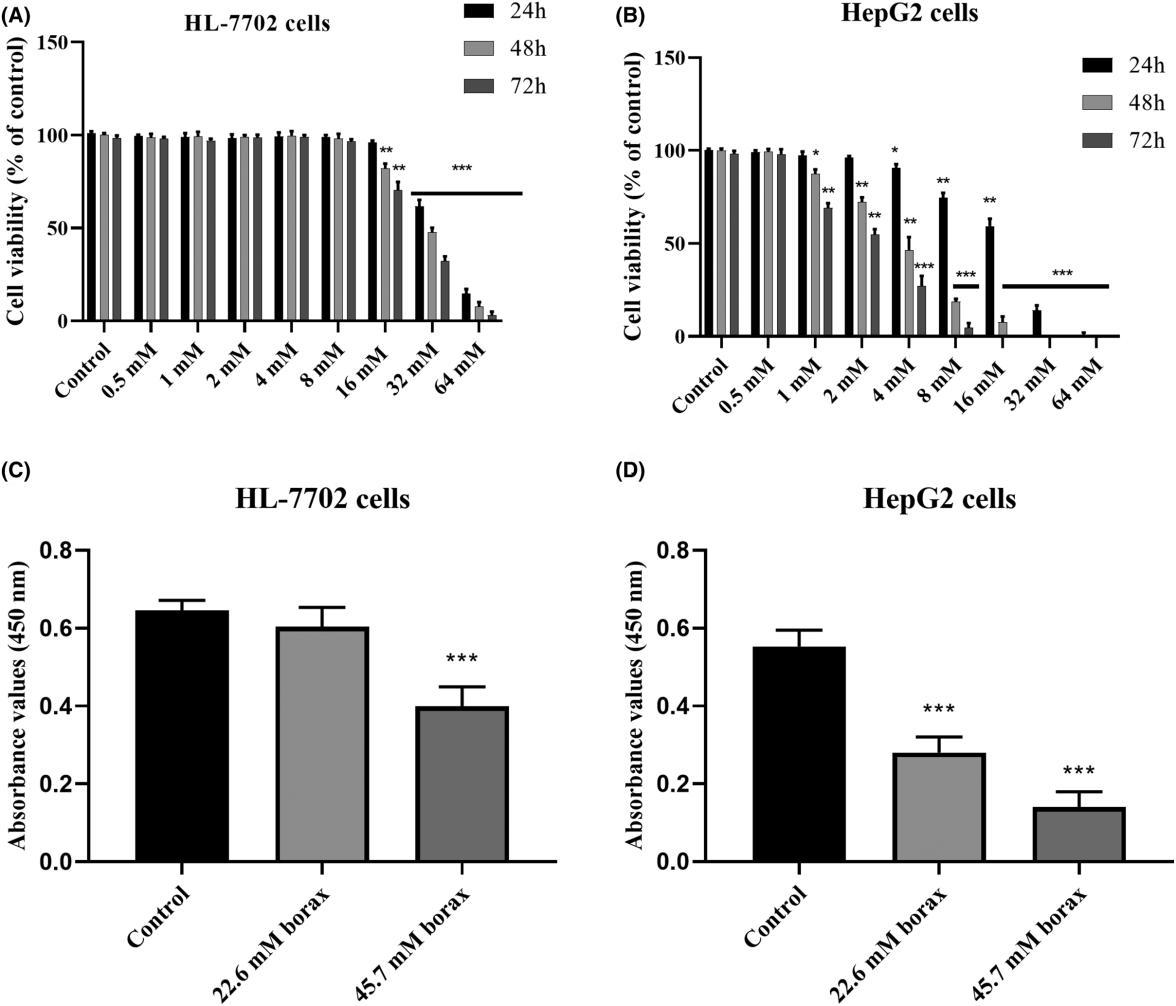
Effects of borax treatment on viability and proliferation of HL‐7702 and HepG2 cells. (A) MTT results in HL‐7702 cells; (B) MTT results in HepG2 cells; (C) BrdU incorporation in HL‐7702 cells; (D) BrdU incorporation in HepG2 cells *p < 0.05, **p < 0.001 and ***p < 0.0001 versus control groups.

Borax regulated cell viability in HepG2 and HL‐7702 cells in a concentration‐ and time‐dependent manner. As shown in Figure 1, HepG2 and HL‐7702 cells were treated with concentrations of 0.5, 1, 2, 4, 8, 16, 32 and 64 mM borax for up to 72 h. Initially, treatment with borax up to a concentration of 32 mM for 24 h and up to 8 mM for 48 and 72 h did not demonstrate a significant impact on the viability of HL‐7702 cells compared to the control group (*p* > 0.05). However, the viability of HL‐7702 cells treated with 32 and 64 mM borax concentrations for 24 h decreased by 38.2% and 83.2%, respectively (*p* < 0.0001 vs. control). Moreover, after treating HL‐7702 cells with borax concentrations of 8 mM and above for 48 and 72 h, the viability of HL‐7702 cells decreased in a concentration‐dependent manner (Figure 1A).

According to MTT results, decreases in the viability of HepG2 cells treated with borax concentrations ranging from 0.5 to 64 mM for 72 h were detected (Figure 1B). The viability of HepG2 cells treated with 0.5, 1 and 2 mM borax concentrations for 24 h did not differ from the control group. However, following treatment with 4, 8, 16, 32 and 64 mM borax concentrations for 24 h, the viability of HepG2 cells decreased by 8.2%, 24.7%, 39.5%, 86.4% and 98.5%, respectively, compared to the control group. (*p* < 0.01 and *p* < 0.0001). Additionally, HepG2 cells viability could not be detected at borax concentrations of 32 mM and above for 48 h and at borax concentrations of 16 mM and above for 72 h. As a result, the 50% inhibitory concentration (IC50) of borax treatment on HepG2 cells for 24 h was 22.6 mM, while the IC50 concentration of borax in HL‐7702 cells was 40.8 mM. According to these results, our analysis revealed that the suppressive effect of borax on cell viability was more pronounced in HepG2 cells compared to HL7702 cells. Based on the MTT results, borax concentrations of IC50 for HepG2 (22.6 mM) and IC75 for HepG2 (45.7 mM) were used during subsequent analysis in HL‐7702 and HepG2 cells.

BrdU analysis was used to determine the effects on cellular proliferation in borax‐treated HL‐7702 and HepG2 cells. Using the IC50 and IC75 values of borax determined according to the MTT results, HL‐7702 and HepG2 cells were treated with 22.6 and 45.7 mM concentrations of borax for 24 h. As seen in Figure 1C, 22.6 mM borax treatment did not cause a significant decrease in the proliferation of HL‐7702 cells compared to the control group, while 45.7 mM borax concentration reduced the HL‐7702 cell proliferation by 43.7% compared to the control group. Additionally, treated with 22.6 and 45.7 mM borax reduced the HepG2 cell proliferation by 55.1% and 82.4%, respectively, in a concentration‐dependent manner (*p* < 0.0001 vs. control; Figure 1D).

### 3.2 Regulation of cell cycle in HL‐7702 and HepG2 cells treated with borax

**FIGURE 2:**
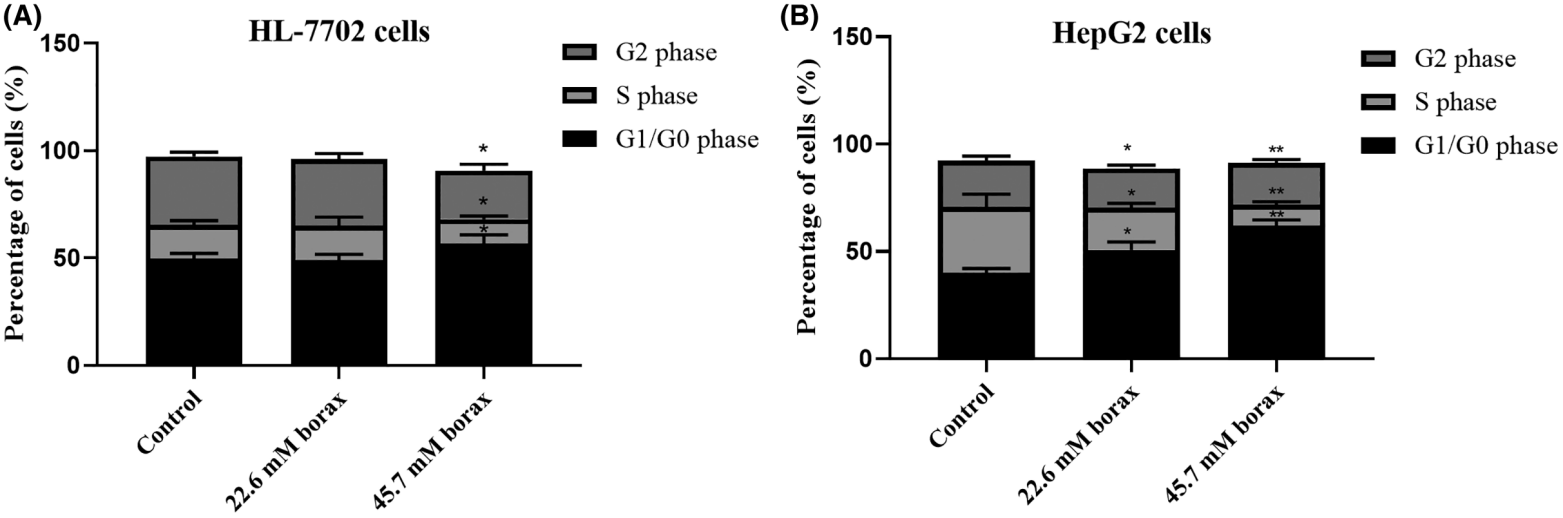
Arresting effects of borax treatment on cell cycle in HL‐7702 and HepG2 cells. (A) Cell cycle analysis in HL‐7702 cells; (B) Cell cycle analysis in HepG2 cells. *p < 0.05 and **p < 0.001 versus control groups.

Figure 2 illustrates the effects of borax treatment on cellular cycles in both HL‐7702 and HepG2 cells. According to flow cytometry analysis results, HL‐7702 cells treated with 22.6 and 45.7 mM borax did not cause a significant change in the G0/G1 and G2 phases compared to the control group, while 45.7 mM borax treatment caused a 10.8% and % 14.6% decrease in the S phase of these cells, respectively, and a 9.3% increase in the G1/G0 phase (*p* < 0.01; Figure 2A). On the other hand, the effects of borax treatment on the cell cycle in HepG2 cells were more pronounced (Figure 2B). Borax treatment decreased the number of cells in the S and G2/M phases and increased the number of cells in the G0/G1 phase in HepG2 cells (*p* < 0.001 vs. control). According to our results, borax treatment arrested HepG2 cells in the G0/G1 phase. In conclusion, while borax had no significant effect on the cell cycle of HL‐7702 cells, it severely suppressed the cell cycle of HepG2 cells.

### 3.3 Borax induced concentration‐dependent morphological differences in both HL‐7702 and HepG2 cells

**FIGURE 3:**
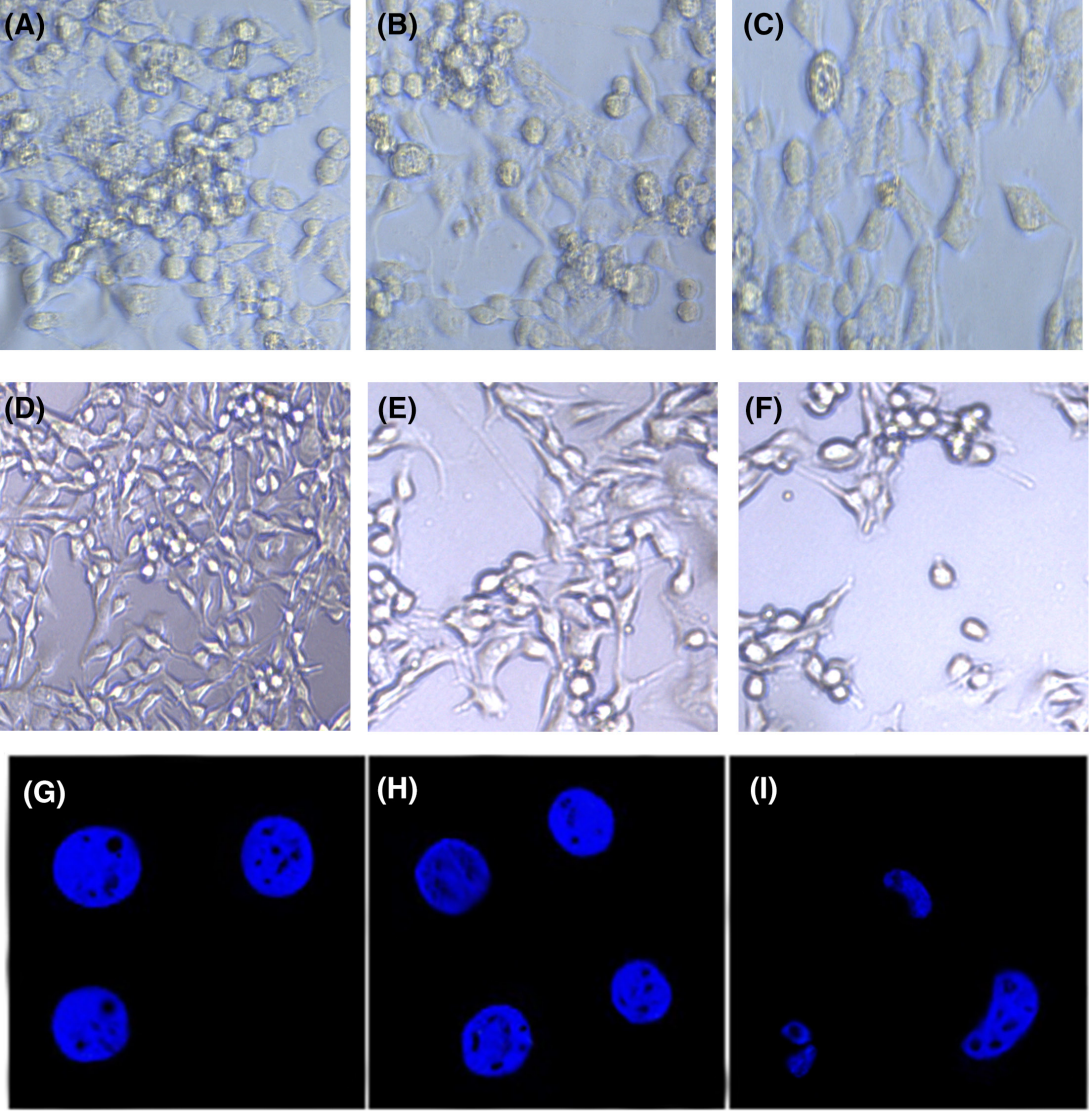
Effects of borax treatment on cellular and nuclear morphologies in HL‐7702 and HepG2 cells. (A–F) Inverted microscope images of HL‐7702 and HepG2 cells; (A) Control group of HL‐7702 cells; (B) 22.6 mM borax treatment group of HL‐7702 cells; (C) 45.7 mM borax treatment group of HL‐7702 cells; (D) Control group of HepG2 cells (E) 22.6 mM borax treatment group of HepG2 cells; (F) 45.7 mM borax treatment group of HepG2 cells; (G–I) DAPI staining of HepG2 cells and scale bars represent 20 μm; (G) Control group of HepG2 cells (H) 22.6 mM borax treatment group of HepG2 cells; (I) 45.7 mM borax treatment group of HepG2 cells. Images in (A–C) (20× objective) and (D‐F) (10× objective).

Cellular morphological abnormalities were examined using inverted microscopy in both HL‐7702 and HepG2 cells following treatment with borax. HL‐7702 cells treated with 22.6 and 45.7 mM borax for 24 h showed no notable abnormalities in cellular size and cytoplasmic volume compared to the control group (Figure 3A–C). However, there are significant differences in both cell number and cell morphology in HepG2 cells treated with 22.6 and 45.7 mM borax for 24 h compared to the control group. Small and round cells with low cytoplasmic content were noticeable in HepG2 cells treated with borax (Figure 3D–F).

Given that borax treatment did not induce morphological abnormalities in HL‐7702 cells, we proceeded to investigate changes in the nuclear structure by conducting DAPI staining specifically in HepG2 cells (Figure 3G–I). Inverted microscopy images revealed morphological distinctions in HepG2 cells. Additionally, DAPI staining was conducted to assess nuclear variances in HepG2 cells. Noteworthy observations included reduced cytoplasmic volume, kidney‐shaped cells, and nuclear abnormalities in HepG2 cells treated with 45.7 mM borax concentration (Figure 3I).

### 3.4 Borax affected SLC12A5, ER‐stress markers and apoptosis markers in both HL‐7702 and HepG2 cells

**FIGURE 4:**
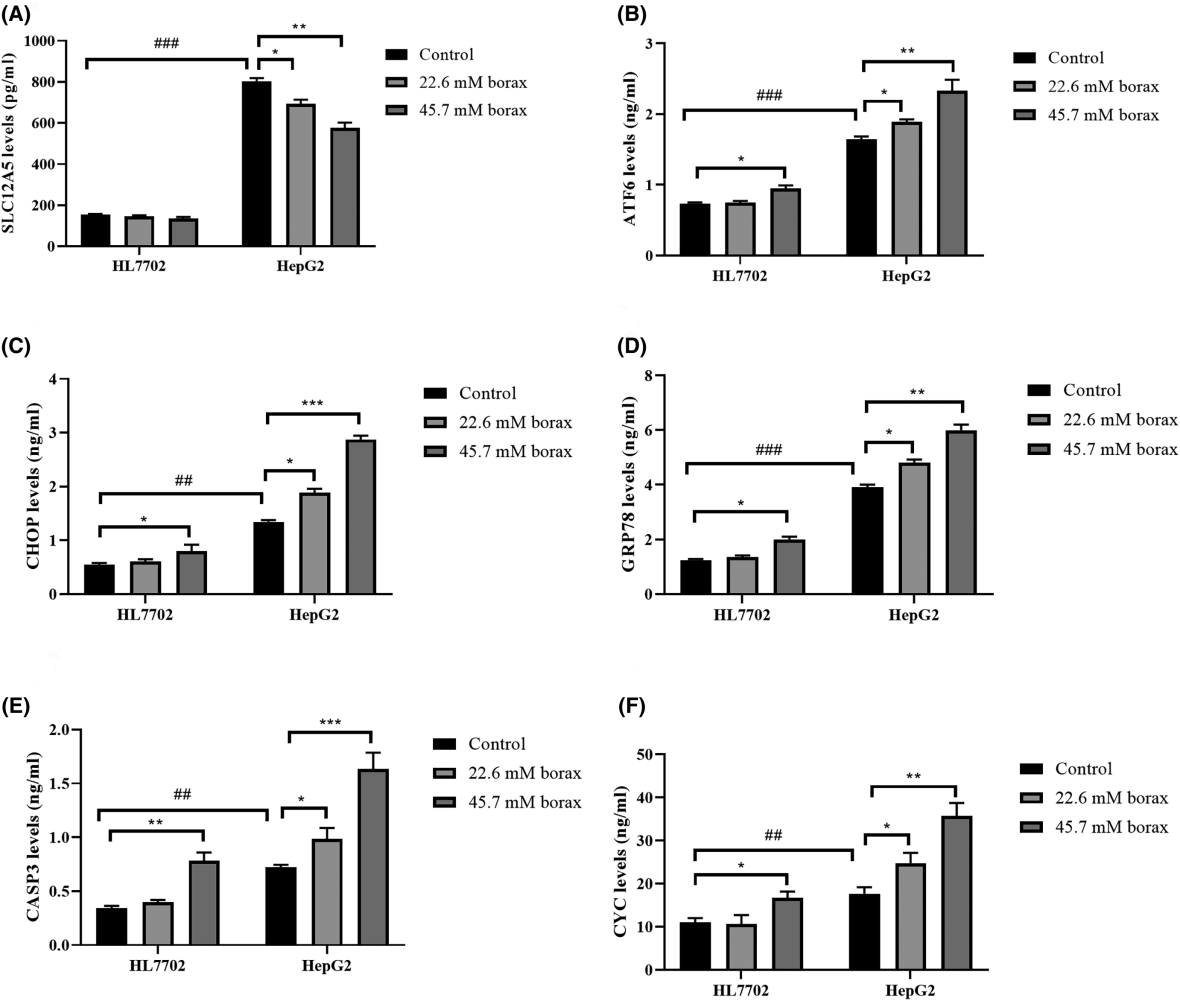
Borax treatment affected SCL12A5, ATF6, CHOP, GRP78, CASP3 and CYC levels in HL‐7702 and HepG2 cells. (A) SCL12A5 levels in HL‐7702 and HepG2 cells; (B) ATF6 levels in HL‐7702 and HepG2 cells; (C) CHOP levels in HL‐7702 and HepG2 cells; (D) GRP78 levels in HL‐7702 and HepG2 cells; (E) CASP3 levels in HL‐7702 and HepG2 cells; (F) CYC levels in HL‐7702 and HepG2 cells. *p < 0.05, **p < 0.001 and ***p < 0.0001 versus control groups. ##*p* < 0.001 and ###*p* < 0.0001 comparison of control groups of HL‐7702 and HepG2 cells.

We investigated SLC12A5 levels in HL‐7702 and HepG2 cells using the ELISA method (Figure 4A). SLC12A5 levels were significantly increased in HepG2 cells compared to HL‐7702 cells (*p* < 0.0001). Distinct differences in SLC12A5 levels were observed in HL‐7702 and HepG2 cells following treatment with borax concentrations of 22.6 and 45.7 mM for 24 h. SLC12A5 levels in HL‐7702 cells showed a modest decrease with borax treatment (*p* > 0.05). However, SLC12A5 levels showed a concentration‐dependent decrease in borax‐treated HepG2 cells (*p* < 0.05 and *p* < 0.001 vs. control).

There were significant differences in ER‐stress‐related ATF6, CHOP and GRP78 levels in HL‐7702 and HepG2 cells (Figure 4B–D). ATF6, CHOP and GRP78 levels in HepG2 cells were higher by 68.2%, 31.4% and 81.5%, respectively, compared to HL‐7702 cells. Moreover, an increase in ATF6, CHOP and GRP78 levels was observed only at 45.7 mM borax concentration in HL‐7702 cells (*p* < 0.05 vs. control). ATF6, CHOP and GRP78 levels in HepG2 cells increased with increasing borax concentration.

Subsequently, we delved into the molecular mechanism through which SLC12A5 and ER stress regulate apoptosis in both HL‐7702 and HepG2 cells (Figure 4E,F). To elucidate the impact of borax on ER stress‐induced apoptosis, we assessed the levels of CYC and CAPS3 in both borax‐treated and untreated HL‐7702 and HepG2 cells. Similar to SLC12A5 and ER‐stress biomarkers, CYC and CAPS3 levels in HepG2 cells were higher than in HL‐7702 cells (*p* < 0.001 and *p* < 0.0001 vs. controls). Simultaneously, CYC and CAPS3 levels increased at 45.7 mM borax concentration in HL‐7702 cells, while CYC and CAPS3 levels showed a concentration‐dependent increase in HepG2 cells.

### 3.5 Effect of borax on SLC12A5, ATF6, CHOP, GRP78 and CASP3 expression and protein levels in HepG2 cells

**FIGURE 5:**
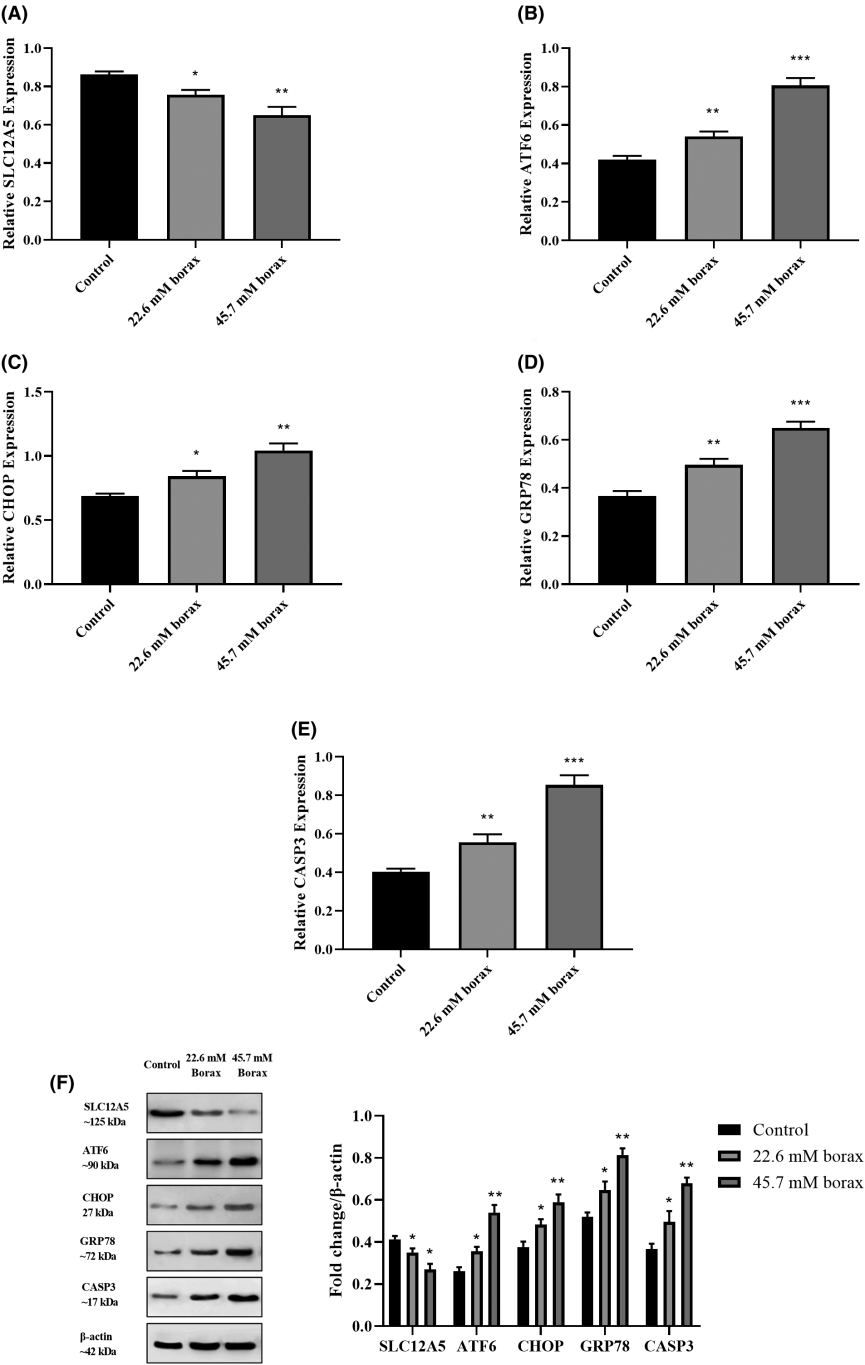
Effects of borax treatment on expression and protein levels of SCL12A5, ATF6, CHOP, GRP78 and CASP3 in HepG2 cells. (A) SCL12A5 mRNA levels in HepG2 cells; (B) ATF6 mRNA levels in HepG2 cells; (C) CHOP mRNA levels in HepG2 cells; (D) GRP78 mRNA levels in HepG2 cells; (E) CASP3 mRNA levels in HepG2 cells; (F) SCL12A5, ATF6, CHOP, GRP78 and CASP3 protein levels in HepG2 cells. *p < 0.05, **p < 0.001 and ***p < 0.0001 versus control group.

The ELISA results revealed variations in SLC12A5, ER‐stress markers, and apoptosis markers between HL‐7702 and HepG2 cells. Notably, it was determined that borax had no significant effect on these biomarkers in HL‐7702 cells. Following these findings, subsequent molecular analyses, such as RT‐PCR and western blotting, were directed towards examining the expression and protein levels of SLC12A5, ATF6, CHOP, GRP78, and CASP3 in HepG2 cells subjected to a 24‐h treatment with borax. In alignment with the ELISA findings, the RT‐PCR results indicated variations in the expression levels of SLC12A5, ATF6, CHOP, GRP78 and CASP3 in HepG2 cells corresponding to the concentration of borax (Figure 5A–E). The expression levels of SLC12A5 in HepG2 cells, treated with borax concentrations of 22.6 and 45.7 mM, were diminished by 11.7% and 22.5%, respectively, compared to the control (*p* < 0.05 and *p* < 0.001). Conversely, the expression levels of ER‐stress biomarkers ATF6, CHOP, and GRP78 in HepG2 cells exhibited an increase in a concentration‐dependent manner. ATF6 expression levels in HepG2 cells treated with 22.6 and 45.7 mM borax concentrations increased by 27.4% and 95.8%, respectively (*p* < 0.001 and *p* < 0.0001 vs. control). Additionally, CHOP expression levels in HepG2 cells increased by 14.25% at 22.6 mM borax concentration and by 41.7% at 45.7 mM borax concentration (*p* < 0.05 and *p* < 0.001 vs. control). Similarly, GRP78 expression levels at 22.6 and 45.7 mM borax concentrations increased by 39.5% and 81.7% compared to the control group, respectively (*p* < 0.001 and *p* < 0.0001). Moreover, we determined that CASP3 expression levels increased by 45.8% at 22.6 mM borax concentration (*p* < 0.001 vs. control) and by 114.2% at 45.7 mM borax concentration (*p* < 0.0001 vs. control) in borax‐treated HepG2 cells, thereby inducing apoptosis.

As depicted in Figure 5F, western blot analysis was employed to assess the protein levels of SLC12A5, ATF6, CHOP, GRP78 and CASP3 in HepG2 cells treated with borax concentrations of 22.6 and 45.7 mM for 24 h. The treatment of HepG2 cells with borax concentrations led to a reduction in SLC12A5 protein levels. Conversely, SLC12A5, ATF6, CHOP, GRP78 and CASP3 protein levels in HepG2 cells exhibited an increase in a concentration‐dependent manner.

## 4 DISCUSSION

Cancer consistently presents a substantial health concern, ranking as the second leading cause of mortality.^32^ Due to the limited efficacy of current treatment modalities, there persists a pressing need for novel therapeutic interventions. HCC, characterized as one of the most high‐risk malignancies, is frequently diagnosed at advanced stages, resulting in restricted treatment options and dismal prognoses.^33^ Consequently, the quest for innovative HCC treatments persists. Boron, an inherently occurring element, has garnered attention for its potential physiological impact on diverse cellular phenotypes and tissues.^34^ Scientific interest in boron compounds has surged, particularly with the acknowledgment of their anti‐carcinogenic attributes. This study endeavours to explore the potential anticancer properties of borax on human HCC cells by evaluating cell viability and proliferation, scrutinizing the cell cycle, and assessing the expression of genes pivotal in apoptosis and ER stress pathways. Furthermore, this investigation elucidates an upregulation in SLC12A5 levels within both cancerous cells (HepG2) and non‐malignant liver cells (HL‐7702). Moreover, the data herein presented posits that SLC12A5, which demonstrates diminished expression in HepG2 cells subjected to borax treatment, may function as an oncogene exerting influence on cell proliferation and the cell cycle.

In the present investigation, MTT assay results unveiled that IC50 of borax for HL‐7702 cells stood at 40.8 mM, whereas for HepG2 cells, it was recorded at 22.6 mM. Correspondingly, it was noted that borax exerted a more pronounced suppression of cell viability in HepG2 cells at lower concentrations. Prior studies have indicated that boric acid, another boron compound, augmented viability in HepG2 cells in a concentration‐dependent manner, exhibiting an IC50 value of approximately 24 mM.^35^ Moreover, in vitro experiments employing various cell lines, such as Ishikawa cells, illustrated a significant decline in cell proliferation at boric acid concentrations of 40 mM and above.^36^ Additionally, our earlier research revealed that treatment with 10.77 mM boric acid in DU‐145 cells led to a noteworthy reduction in cell growth.^37^ Collectively, extant literature delineates a trend wherein boron compounds impede cancer cell proliferation beyond a certain concentration threshold. Boron neutron capture therapy (BNCT) is a radiotherapy method that uses tumour‐targeting boron (^10^B) drugs such as boronophenylalanine (BPA) and sodium borocaptate (BSH) to trigger tumour‐specific damage without damaging surrounding normal cells.^38^ A recent study showed that boric acid may be a suitable agent for BNCT specifically in HCC.^30^ Additionally, in this study using an in vivo HCC model using boric acid‐mediated BNCT, it was shown that tumour blood vessels absorb much higher levels of boric acid than normal liver tissues, thus disrupting blood flow in the tumour tissue and accelerating the death of tumour cells. Furthermore, previous reports indicate that BNCT treatment engenders diminished cell growth, disruption of the cell cycle, disruption in DNA integrity and chromosomal organization, alongside substantial alterations in the transcriptomic profile in radiotherapy‐resistant human HCC cells.^39^ In consonance with these findings, our study observed, through microscopic examination, that borax treatment induced specific morphological and nuclear aberrations in HepG2 cells.

Our findings underscore SLC12A5 as a potential oncogene governing proliferation, cell cycle advancement, ER stress, and apoptosis in HCC. Physiologically, heightened SLC12A5 expression in HepG2 cells, relative to HL‐7702 cells, fosters cellular proliferation by extending the S phase of the cell cycle via intracellular ER stress induction. Subsequently, borax treatment exhibited limited effects on cell viability and proliferation in HL‐7702 cells while demonstrating anti‐proliferative impacts in HepG2 cells. Notably, borax treatment activated ATF6/CHOP/GRP78 signalling, further emphasizing its suppressive actions on tumorigenesis. Accumulating evidence from both in vitro and in vivo investigations in the literature corroborates the oncogenic properties ascribed to SLC12A5. For instance, studies propose that SLC12A5 may bolster bladder cancer progression through various signalling pathways, including nuclear factor kappa B, matrix metalloproteinase‐7, and SOX18.^40^ Additionally, it has been found that SLC12A5 impedes tumorigenesis in HepG2 cells by impeding cell cycle progression via calcium release into the cytosol during ER stress.^20^ While conventionally recognized as a membrane protein crucial for K‐Cl transport, recent evidence unveils that SLC12A5 is also expressed in the nucleus, exerting a tumour‐promoting role.^41^ Jiang and colleagues conducted a comprehensive investigation into the role of SLC12A5 across 33 human cancers, demonstrating its potential as a tumour promoter associated with worse overall survival and poor disease‐specific survival.^42^ Additionally, Wei and et al. reported that metastatic activities in cervical cancer cells can be induced via the SLC12A5 mechanism.^43^ These results suggest the potential utility of SLC12A5 as a prognostic biomarker in certain malignancies. Consistent with the aforementioned studies, the overexpression of SLC12A5 in HepG2 cells compared to HL‐7702 cells facilitated cell cycle progression, as revealed by flow cytometry analysis focusing on the cell cycle. Simultaneously, the reduction of SLC12A5 expression levels through borax treatment led to the arrest of malignant proliferation in the S phase in HepG2 cells.

The ER stress response in cancer cells serves to minimize damage and has recently been implicated in various cell death pathways, including ferroptosis, tumorigenesis, apoptosis and autophagy.^44^ However, the specific signalling mechanisms of ER stress in borax‐treated HCC are not yet fully understood. GRP78 and CHOP, pivotal components in ER stress, play crucial roles in initiating and amplifying the signalling cascade during ER stress activation.^45^ Under normal physiological conditions, GRP78 resides in the ER lumen bound to transmembrane proteins such as PERK, IRE1 and ATF6. Upon induction of ER stress, GRP78 dissociates from these proteins and activates ER stress signalling.^46^ Additionally, CHOP, another ER stress marker, assumes a pro‐apoptotic role in the stress response.^47^ In the context of boron compounds, a previous study involving boric acid demonstrated an increase in eIF2α phosphorylation and BiP/GRP78 translation in DU‐145 cells.^48^ This study also revealed that boric acid induced mild ER stress along with a slight activation of the eIF2α/ATF pathway, suggesting that physiological concentrations of boric acid may promote the anticarcinogenic effects of boron. Tong and colleagues reported that SLC12A5 promotes tumour progression by regulating ER stress and increases ferroptosis resistance in HCC by controlling intracellular redox balance.^20^ In this study, our investigation focused on the gene expressions of biomarkers related to ER stress pathways induced by borax in HepG2 cells. Additionally, we explored the connection between borax‐induced ER stress and apoptotic mechanisms, as well as its impact on SLC12A5. Our findings indicate a significant escalation in ER stress, as evidenced by heightened expression and protein levels of GRP78, CHOP and ATF6 in borax‐treated HepG2 cells. Furthermore, the intensified ER stress induced by borax treatment correlated with an increase in CASP3 levels, suggesting an association with apoptosis. Conversely, SLC12A5 expression levels decreased in borax‐treated HepG2 cells, indicating a contrasting response wherein ER stress and apoptosis were induced. These findings uncover the association between ER stress and borax in HepG2 cells and shed light on the oncogenic effects of SLC12A5 in HCC, as well as the influence of borax on these effects.

## 5 CONCLUSION

In summary, our study elucidates the significant role of SLC12A5 in regulating metabolic functions in hepatocellular carcinoma (HCC). The comparatively lower levels of SLC12A5 observed in HL‐7702 cells in contrast to HepG2 cells suggest its potential contribution to cellular survival in HCC. Moreover, borax treatment was observed to induce ER stress by downregulating SLC12A5 levels in HepG2 cells. This resulted in a notable increase in the expression levels of ER stress markers GRP78, CHOP, and ATF6, ultimately diminishing the viability of HepG2 cells. Additionally, we noted that the heightened ER stress induced by borax treatment coincided with apoptosis. However, it is essential to acknowledge that our study lacks in vivo validation, underscoring the necessity for further investigation to validate and refine our findings. Our results contribute to a deeper understanding of the multifaceted role of boron compounds in HCC treatment. Specifically, our findings highlight the capacity of borax to modulate ER stress through the SLC12A5 signalling pathway. Further research endeavours are warranted to validate and expand upon the implications of our observations.

## AUTHOR CONTRIBUTIONS

**Ceyhan Hacioglu:** Conceptualization (lead); data curation (lead); formal analysis (lead); funding acquisition (lead); investigation (lead); methodology (lead); project administration (lead); resources (lead); software (lead); supervision (lead); validation (lead); visualization (lead); writing – original draft (lead); writing – review and editing (lead). **Didem Oral:** Formal analysis (supporting); investigation (supporting); methodology (supporting).

## FUNDING INFORMATION

None.

## CONFLICT OF INTEREST STATEMENT

The authors have no relevant financial or non‐financial interests to disclose.

## Data Availability

The datasets generated from the corresponding author on reasonable request.
